# Cerulean cataract

**DOI:** 10.11604/pamj.2023.45.16.39806

**Published:** 2023-05-05

**Authors:** Meryem Benchekroun Belabbes, Narjisse Taouri

**Affiliations:** 1 Mohammed V Souissi University, Faculty of Medicine and Pharmacy, Rabat, Morocco

**Keywords:** Cerulean, cataract, visual acuity

## Image in medicine

We report a case of a 34-year-old woman with a medical history of diabetes type 1 presented to our ophthalmology department due to a progressive diminution of vision in both eyes during the last year. No history of ocular trauma was reported. Her best corrected visual acuity was 20/50 in both eyes. Slit lamp examination of the lenses of both eyes revealed white tiny opacities forming concentric layers and a dense posterior subcapsular cataract (A, B). The posterior segment was unremarkable in both eyes with no sign of diabetic retinopathy. The patient underwent cataract surgery of the right eye and three months later of the left eye. The two surgeries proceeded without complications with a final visual acuity of 20/20 for both eyes. Cerulean cataract is a rare form of congenital cataract which usually presents as bilateral, blue-white opacities in the lens arranged in concentric layers. Armitage MM *et al*. reported that it is a genetically inherited disease transmitted in an autosomal dominant pattern. Multiple causative mutations have been identified, including mutations in the beta-B2-crystallin gene (CRYBB2), gamma-D-crystallin gene (CRYGD), V-MAF avian musculoaponeurotic fibrosarcoma oncogene homolog gene (MAF), the major intrinsic protein of lens fiber gene (MIP). The treatment is surgical and consists of the removal of the cataract, if the visual acuity is reduced, with the placement of an artificial lens.

**Figure 1:**
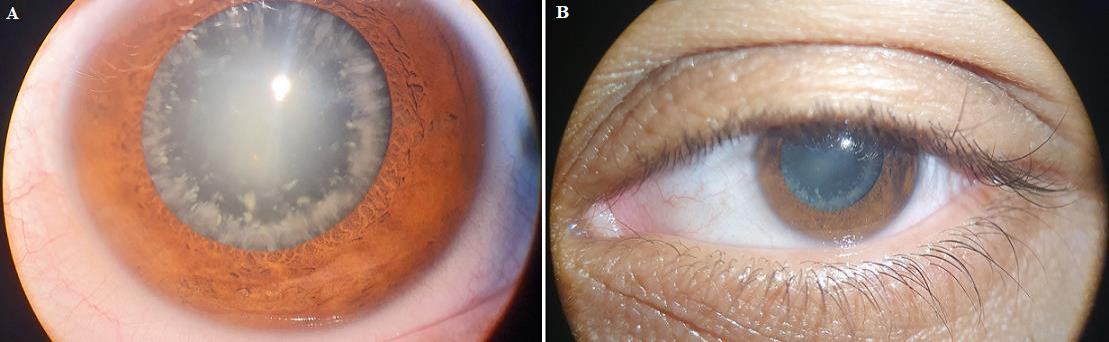
A) slit lamp photo of cerulean cataract of the right eye; B) slit lamp photo of cerulean cataract of the left eye

